# It Is the Time to Treat Endometriosis Based on Pathophysiology

**Published:** 2019

**Authors:** Shahla Chaichian

**Affiliations:** - Minimally Invasive Techniques Research Center in Women, Tehran Medical Sciences Branch, Islamic Azad University, Tehran, Iran; - Pars Advanced and Minimally Invasive Medical Manners Research Center, Pars Hospital, Iran University of Medical Sciences, Tehran, Iran

According to the literature, endometriosis was first explained in Egyptian scrolls in the sixteenth century BC (1). But the disease remained unknown in many human societies and patients underwent inappropriate treatment from incomplete drowning to even burning at the shadow of this belief that Satan has conquered their soles.

Although Carl Freiherr von Rokitansky described the scientific and histological aspects of endometriosis in 1860 (2), the disease was considered as an ambiguous disorder along with unclear pathology till about 1.5 century later (1).

Despite this astonishing description, mismanagement of the endometriosis, continued to the extent that even Freud misdiagnosed the individuals with the disorder as hysteric patients and tried to treat them by hypnosis in the last decades of 19 th and the first decades of the 20 th century.

For the first time, the term “endometriosis” was issued by Sampson in 1925 (3–5). With the continuation of his studies, his brilliant paper based on retrograde menstruation theory as a cause of peritoneal endometriosis was published in 1927 (5). He also noticed the higher incidence of endometrioid and clear cell carcinoma in the endometrioma (1). It seems that the number of individuals with the risk of ovarian cancer approaches to 7 times greater than the normal population for these isolated types. So far according to Nezhat, the disease can be considered as a kind of screening tool for ovarian cancer (6).

Georg Kelling carried out the first laparoscopic surgery on dogs in 1902. In addition, Hans Christian Jacobaeus applied the approach to operate on a human being in 1910. However, the procedure was modified and popularized by some pioneer people during the next couple of decades (7, 8).

At that time, surgeons were performing many kinds of surgeries on these women especially oophorectomy in the hope of overcoming symptoms, and reducing the risk of infertility and ovarian cancer.

Over time and in the shadow of technical and instrumental progress in laparoscopy, this technique turned to the gold standard manner for the treatment of endometriosis.

As the time passed, it was cleared that, laparoscopic surgery is not the final solution for all endometriosis patients, and even worse, in some cases induces premature ovarian insufficiency and/or poor response to ART techniques. This is far beyond internal complications and hazards every surgery carries.

Simultaneously, our insight into the pathophysiology of endometriosis were deerened through large unmber of studies. Based on the current evidence, various etiopathological factors such as genetic, epigenetic, environmental, immunological, stem cells and/or endocrine processes are involved. So far, no specific susceptibility genes have been identified. Endometriosis is indeed a benign disorder (9).

The hormonal aspect of endometriosis for many years has helped physicians to treat endometriosis. However, these numerous products have their own complications and restrictions, especially at the time of pregnancy desire and in menopausal patients.

In recent years, researchers have founded more sophisticated etiopathogeneses for the disease. According to a recent study, extrauterine stem cells originating from bone marrow are able to differentiate into endometriotic tissues. Despite confirmation of the role of molecular medicine as a new method for clarifying the pathogenesis of endometriosis, Sampson’s implantation theory has never been discarded (1).

Jones in 1998 indicated that the women with endometriosis reveal upregulation of the antiapoptotic gene BCL-2 in eutopic and ectopic endometrium. Moreover, he postulated that genetic alterations of endometrial cells which impact their tendency to implant may be hereditary (10). Linkage analysis done by Treloar in 2005 has revealed candidate genes with biological plausibility, as well (11).

The hypothesis of endometriosis as a pelvic inflammatory condition is accepted. In these patients, the peritoneal fluid and the number of activated macrophages are increased significantly. There is also a great variation in the cytokine/chemokine profile. Macrophages can produce and secrete various biologically active elements (*i.e*. cytokines, plasma proteins, coagulation and fibrinolytic agents, enzymes components of complement, and lipids). A unique protein similar to haptoglobin has been discovered by proteomics method in the peritoneal fluid of endometriosis patients (12). This protein decreases macrophage phagocytic activities after bounding process and enhances production of IL-6. Macrophage migration inhibitory factor, TNF-α, IL-1β, IL-6, and IL-8, and monocytes are in the peritoneal fluid of endometriosis patients (1).

Integrin and E-cadherin and MMPs namely ICAM (Intracellular adhesion molecules) have been discovered in cells of menstrual effluent, endometrium, peritoneal fluid, peritoneum, and endometriotic cells (13).

Langendonckt in 2002 showed a significant role of hemoglobin in the pathogenesis of peritoneal endometriosis. This hypothesis confirms that, after RBC lysis, the resultant released hemoglobin in the peritoneal cavity induces activation of cell adhesion molecules, cytokine production, cell proliferation, and neovascularization. Hemoglobin molecules degradation to its product, heme, Iron, biliverdin and bilirubin may lead to oxidativ stress (14).

Despite these and other progresses in understanding the pathophysiology of this enigmatic disease and many compounds that their effectiveness has been postulated in several clinical trials, some, as gynecologic laparoscopists are continuing to transfer their habitual concepts that laparoscopy is the best treatment for endometriosis.

With regard to ACOG guideline and much more scientific evidence, now it is the time to treat this malefic disease based on these causative etiologies and reserve surgery for the most important occasion during patient’s lifetime.
